# Constructing Stable Emulsion Gel from Soy Protein Isolate and Konjac Glucomannan as Pork Fat Substitute: Effect of Oil Concentration

**DOI:** 10.3390/foods14162760

**Published:** 2025-08-08

**Authors:** Junjie Tang, Danjie Li, Jianxi Zhang, Xianyang Xie, Si Shi, Jie Shi, Yuanzhao Li, Jie Pang, Chunhua Wu

**Affiliations:** 1 Engineering Research Centre of Fujian–Taiwan Special Marine Food Processing and Nutrition, Ministry of Education, College of Food Science, Fujian Agriculture and Forestry University, Fuzhou 350002, China; junjietang0907@163.com (J.T.); 15216948080@163.com (D.L.); 13387399569@163.com (J.Z.); 18905973317@163.com (X.X.); 15059760207@163.com (S.S.); 18506028958@163.com (J.S.); pang3721941@163.com (J.P.); 2 Coll Equipment Management & Supportabil, Engineering University of Peoples Armed Police, Xi’an 710086, China; leeyz1234@163.com

**Keywords:** emulsion gels, animal fat analog, corn oil, soy protein isolate, konjac glucomannan

## Abstract

Vegetable oils offer a healthy option for creating nutritious foods. This research created a plant-based fat substitute inspired by nature. We used soy protein isolate (SPI), which acts both as a base material and an emulsifier. To strengthen the structure, we added oxidized chitin nanowhiskers. We mimicked the texture of animal fat by forming a gel using konjac glucomannan (KGM). By fine-tuning the corn oil ratio, we developed a dense, structurally superior emulsion gel. Gels containing 12% corn oil demonstrated effective water and oil retention, with their texture, gel strength, hardness, and chewiness closely resembling that of pork back fat. It also matches the visual properties of pork back fat in terms of brightness and hue. However, it offers significant health benefits over pork back fat, as it contains significantly lower fat content, only 14.07% of that of pork back fat, predominantly healthier unsaturated fats, and a protein content 2.74 times higher than that of pork back fat. This KGM/SPI gel system provides a practical, scalable way to develop solid fat alternatives that are both sustainable and rich in nutrients, supporting the creation of healthier foods for the next generation with a reduced fat content.

## 1. Introduction

Fat is a crucial flavor carrier in meat products, but its high content of unhealthy saturated fats is linked to obesity, colorectal cancer, and cardiovascular diseases [1,2,3]. Consequently, the meat industry aims to reduce animal fat to develop healthier products [4]. However, this reduction compromises key qualities like moisture retention, flavor, and aroma, impacting consumer acceptance [5]. Therefore, achieving a balance between reducing fats and maintaining the desirable qualities of meat products remains a critical focal point for ongoing research and innovation.

Vegetable oils are gaining attention as healthier alternatives to animal fats due to their favorable fatty acid profiles and cholesterol-lowering potential. Corn oil, rich in unsaturated fatty acids (oleic, linoleic, linolenic), vitamin E, polyphenols, and squalene, helps prevent LDL cholesterol accumulation [6]. Linoleic acid is appreciated for its potential to prevent cardiovascular and cerebrovascular diseases while offering antioxidant and anti-aging benefits. However, substituting animal fats with vegetable oils, which have lower melting points, poses some challenges. These oils can alter the flavor and compromise the processing quality of meat products. This necessitates modifications to the structure of vegetable oils to emulate the texture and plasticity of solid fats, ensuring that substitutes do not compromise the product’s quality [7].

Emulsified gels solidify unsaturated vegetable oils by dispersing droplets within a continuous gel network, imparting solid-like properties. In protein-based water-in-oil emulsions, gel networks form in the aqueous phase through acid/heat/enzyme-induced gelation or polysaccharide addition, stabilizing droplets and creating solidity [8]. Soy Protein Isolate (SPI) is widely used for emulsion gels due to its nutrition and emulsification [9], but its weak gelation limits its mimicry of solid fats. Incorporating polysaccharides enhances protein flocculation, with protein–polysaccharide ratio adjustment improving gel performance.

In the research of SPI emulsion gels, Konjac Glucomannan (KGM) serves as a popular polysaccharide. Under alkaline heating conditions, this method hydrolyzes the ester bonds on KGM chains to remove acetyl groups, which allows the glucomannan chains to form hydrogen bonds, creating a gel effect similar to pork back fat [10]. For instance, [11] developed a complex fat substitute that combines soybean isolate protein with coconut oil, to which konjac flour was added to replicate the elasticity, texture, mouthfeel, and lubricity of natural fat. Similarly, [12] created a plant-based cubic fat substitute, comprising a composite emulsion gel of SPI and KGM, and used it to produce a well-accepted, low-fat pork patty. Additionally, introducing nanofibers such as Oxidized chitin nanofibers (O-ChNWs) as fillers has been proven to effectively enhance and stabilize KGM gels in our previous studies [13].

This study examines the influence of corn oil concentration gradients on the rheological properties, water-holding capacity, and oil-holding capacity of emulsion gels. The structural properties of the gels were comprehensively characterized through multi-modal analytical spectroscopy (Fourier transform infrared spectroscopy/X-ray diffraction) and advanced microscopy techniques (Scanning electron microscope/Confocal laser scanning microscopy). In addition, the effects of the corn oil concentration gradient on the appearance, color, texture, microstructure, and nutrient content of emulsified gels were elucidated. A comparative evaluation framework was established to assess the techno-functional parity between fat analogs gels and conventional porcine adipose tissue, focusing on mechanical properties, chromatic characteristics, macronutrient composition, and energy density profiles. These insights advance the scientific foundation for developing healthier, low-fat meat products.

## 2. Materials and Methods

### 2.1. Materials

O-ChNWs were prepared in previous studies [13] with a yield of 35% based on the initial weight of chitin. SPI purchased from Linyi Shansong Biological Products Co., Ltd. (Linyi, China). KGM (purity > 90%) was purchased from Shanghai Beilian Biological Technology Co., Ltd., (Shanghai, China). All other reagents used were of analytical grade.

### 2.2. Preparation of Emulsion Gels

**Figure 1:**
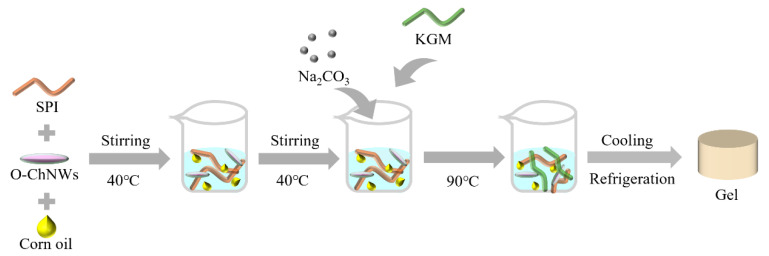
Schematic diagram of emulsion gel formation.

To prepare the samples, 0.225% O-ChNWs were dispersed using 50% ultrasonic power for 10 min. The 14.5% SPI powder was then dissolved in deionised water. Varying concentrations of corn oil (0%, 4%, 8%, 12%, 16%) were incorporated into the mixture, which was mechanically stirred at 500 rpm in a 40 °C water bath for 90 min to ensure proper dispersion and hydration. Subsequently, 0.45% Na_2_CO_3_ solution was added while increasing the stirring speed to 650 rpm for another min. Then, 1.5% KGM was gradually added and this was constantly stirring for one minute to form a sol. This sol was then heated in a 90 °C water bath for 90 min. After heating, the gel was cooled to room temperature over one hour and refrigerated at 4 °C for 12 h to set. Prior to further analysis, the gels were equilibrated at room temperature for one hour. The samples were labeled SKOC, SKOC4, SKOC8, SKOC12, and SKOC16 for clarity in subsequent evaluations. Figure 1 shows a schematic diagram of the formation of an emulsion gel.

### 2.3. Rheological Properties

The mechanical characteristics of the gel solution prior to heat treatment were analyzed with a rheometer. The examination of how viscosity correlated with shear rate involved conducting measurements from 0.1 to 100 s^−1^ at a controlled temperature of approximately 25 °C.

Using strain values between 0.01% and 100% and a frequency of 1 Hz, the linear viscoelastic region of the samples was determined. Subsequently, within this linear viscoelastic domain, an angular frequency sweep test was performed using a strain amplitude of 0.5%. Angular frequency sweeps were tested for frequency values ranging from 1 to 100 rad/s.

### 2.4. Fourier Transform Infrared Spectroscopy (FT-IR)

Characterization of the freeze-dried specimens was conducted using Fourier Transform Infrared Spectroscopy with a Bruker VERTEX 70 device (Thermo Fisher Scientific Co., Ltd., Waltham, MO, USA). These analyses were performed at a constant temperature of 25 °C, covering a spectral range from 4000 to 400 cm^−1^.

### 2.5. X-Ray Diffraction (XRD)

The emulsion gel sample was placed on a tray and an X-ray diffractometer (D8 ADVANCE, Bruker, Salbuluken, Germany) was used to obtain the X-ray diffraction spectrum. The diffraction measurements covered an angular range of 5 to 70° in 2θ, with the scanning rate set at 2° per min.

### 2.6. Scanning Electron Microscope (SEM)

The freeze-dried gel specimens, after being coated with gold through sputter deposition, were examined under an electron microscope. This analysis was performed at a magnification of 50× and an acceleration voltage set at 5.0 kV, aiming to observe the microstructural details of the samples.

### 2.7. Confocal Laser Scanning Microscopy (CLSM)

The methodology proposed by [14] was adapted to study the distribution of oil droplets within gels by employing a confocal laser scanning microscope (LSM710, Carl Zeiss, Oberkohen, Germany). Each sample was sectioned into 1 mm slices and stained for 40 min using Nile blue (0.4%, *w*/*v*) for proteins and Nile red (0.1%, *w*/*v*) for lipids. After staining, the samples were rinsed with distilled water to remove excess dye and subsequently examined under a 20× optical microscope.

### 2.8. Water Holding Capacity (WHC) and Oil Binding Capacity (OHC)

The protocol was adjusted from the approach suggested by [15], aimed at evaluating the water and oil retention capacities of the gel. For the WHC assessment, a 2 g sample of the gel was cooled to 4 °C for 24 h and subsequently allowed to stabilize at ambient temperature. The sample then was placed into a 10 mL centrifuge tube and spun at 3000 rpm for 5 min at 25 °C. Any additional moisture was absorbed using filter paper before and after the sample was weighed. WHC was calculated by comparing the moisture content pre- and post-centrifugation. For OHC evaluation, a different 2 g sample was heated in a water bath at 70 °C for 30 min, followed by centrifugation at 6000 rpm for 15 min. The expelled oil was gathered in a pre-weighed crucible and left to dry at 100 °C overnight. The change in weight of the crucible indicated the OHC.

### 2.9. Appearance and Color Determination

Images of the emulsion gel were captured using a smartphone. The color of the emulsion gel is determined by a colorimeter (WSC-100, Beijing Optical Instrument Factory, Beijing, China).

### 2.10. Texture Profile Analysis (TPA)

For texture analysis, a texture analyzer was employed in TPA mode, utilizing a P/36 probe. The operational settings were consistent across all phases: the pre-test, test, and post-test speeds were set at 1.0 mm/s, with a trigger force of 5 g, and a deformation target of 40%. To measure the gel strength, the same analyzer equipped with a P/5 probe was used. The settings remained the same for the speed and trigger force, with the compression distance fixed at 10 mm.

### 2.11. Nutrient Content Determination and Energy Values

Zhao et al. [16] reported that the levels of protein, fat, moisture, and ash in emulsion gels and pork back fat were quantitatively analyzed. The caloric content of these samples, expressed in kilojoules (kJ), was determined using conversion rates of 17 kJ/g for protein and 37 kJ/g for fat, which were set forth by Kang et al. [17].

### 2.12. Statistical Analysis

Each measurement was conducted a minimum of three times, and the results are shown as the average ± standard deviation. The data underwent analysis through the SPSS 26.0 software, utilizing a one-way analysis of variance (ANOVA) with a significance threshold of *p* < 0.05. Subsequent analysis included Duncan’s multiple range test to further examine the differences.

## 3. Results and Discussion

### 3.1. Rheological Properties of Emulsion Gels

**Figure 2:**
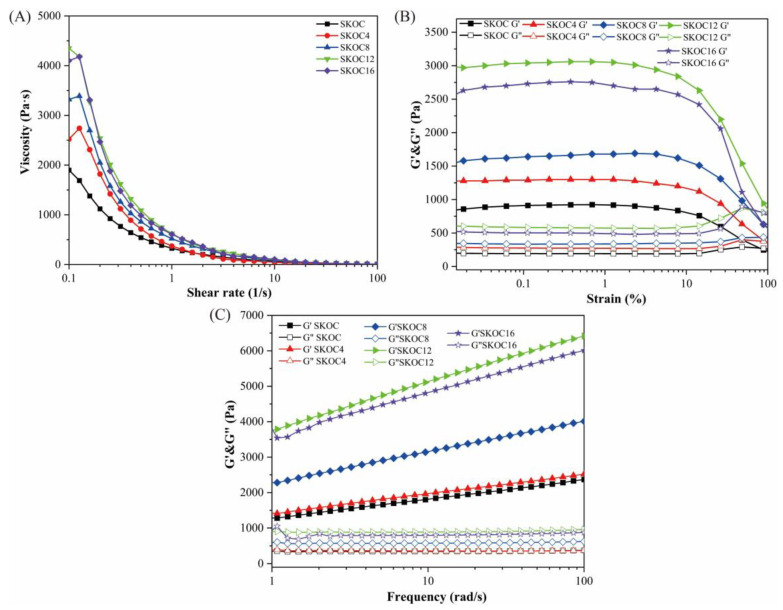
Apparent viscosity (**A**), amplitude oscillatory sweeps (**B**), frequency sweeps (**C**) and rheological properties of emulsion gels with different oil fractions.

Apparent viscosity is an important index for characterizing the stability of gels; it is used to describe the flow properties of fluids and reflects the difficulty of disrupting the internal structure of the gel. Figure 2A illustrates that as the shear rate increases, the viscosity consistently decreases across all samples, exhibiting a “shear-thinning” phenomenon. High shear rates can lead to the deformation of agglomerated droplets and the breakdown of the network structure, thereby lowering viscosity by reducing flow resistance. Compared to the SKOC system, the addition of corn oil to the composite hydrogel results in higher viscosity. This stability enhancement is attributable to corn oil, which organizes and stabilizes the composite’s structure, making it more resistant to shear-induced alignment and thus increasing its viscosity [18,19]. According to Figure 2A, the network structure formed by SKOC12 is the most difficult to disrupt, indicating the presence of the strongest interactions in this system. When the corn oil content in the composite hydrogel surpasses 12%, no additional increase in apparent viscosity is noted, suggesting that the system’s viscosity stabilizes once the oil concentration reaches this threshold.

Under variable strain amplitude conditions, we determined the linear viscoelastic state of the gel. Within a strain range of 0–100%, both the storage modulus (G′) and loss modulus (G″) stayed consistent. However, as the strain amplitude increased, evidenced by Figure 2B, both G′ and G″ began to decrease, culminating in a crossover—suggestive of a shift from a gel-like state to a viscosity-dominated structure, which indicates irreversible damage to the gel network structure [20].

Figure 2C illustrates the variation in the G′ and G″ of SKOC gels with angular frequency at different corn oil concentrations. The frequency dependence of the viscoelastic parameters is crucial for assessing the gel’s structural integrity. Across all tested frequencies, the G′ values exceeded G″ values, indicating that the gel system exhibited typical solid-dominated behavior with pronounced gel characteristics. Notably, the G′ and G″ curves showed no crossover within the tested frequency range, confirming the robust mechanical properties of the emulsion gel formed within the linear viselastic region [21]. The underlying mechanism involves SPI forming a continuous gel matrix that collaboratively constructs a stable network with KGM via hydrogen bonding [22]. Simultaneously, oil droplets are uniformly embedded within this network, collectively forming a high-strength gel structure [23]. Similar phenomena have also been observed in the study by Su et al. [24]. However, when the oil concentration increased to 16%, the excess oil exceeded the network’s embedding capacity, leading to oil droplet aggregation and the disruption of structural homogeneity [25]. This resulted in a significant decrease in G′ and G″. This observation aligns with the findings of Jiang et al. [26], indicating that a high oil content compromises the structural stability of emulsion gels and consequently reduces their rheological moduli.

### 3.2. FT-IR Analysis of Emulsion Gels

**Figure 3:**
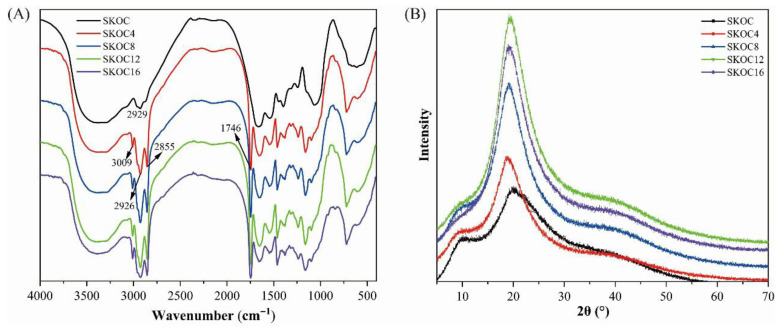
The FT-IR spectra (**A**) and XRD profile (**B**) of emulsion gels.

Figure 3A displays the FT-IR spectra of gels, all demonstrating similar peak profiles, indicative of consistent structural characteristics. SPI, known for its complex biomolecular structure, presents defined infrared absorption bands prominently in the amide I and II regions. Specifically, the amide I band, located between 1600 and 1700 cm^−1^, corresponds to C-O and C-N stretching vibrations [27], while the amide II band, occurring from 1510 to 1550 cm^−1^, is associated with N-H bending and C-N stretching. Additionally, the broad bands observed between 3700 and 3200 cm^−1^ indicate the presence of free and hydrogen-bonded O-H and N-H vibrations, suggesting significant inter- and intra-molecular hydrogen bonding [28]. The spectra also highlight peaks at 3009 cm^−1^, 2925 cm^−1^, and 2854 cm^−1^, which are attributable to C-H stretching vibrations. Notably, shifts in these peaks are observed as the oil concentration increase beyond 12% (*v*/*v*), possibly signifying that excess oil aggregates may disrupt hydrogen bonding and diminish the network’s uniformity.

A distinct peak at 1746 cm^−1^ is introduced with the addition of corn oil, attributed to the stretching vibrations of the carbonyl C=O group in the triglycerides from the oil [29]. These findings suggest that corn oil contributes more than just filling effects; it likely participates in subtle interactions with the gel’s proteins or polysaccharides, altering peak positions in the spectra.

### 3.3. XRD Analysis of Emulsion Gels

XRD is an effective tool for analyzing the crystalline structures or morphologies of materials, with a direct positive correlation between crystallinity and the sharpness of diffraction peaks (Figure 3B). Higher crystallinity yields more defined peaks [30,31]. All emulsion gels samples displayed a broad peak around 2θ = 20°, indicating that all gels were in an amorphous state [32,33]. The diffraction patterns of SKOC and oil-containing gels were almost the same. Minor differences lie in the sharpness and intensity of the main diffraction peaks. Notably, as the concentration of corn oil increases, the diffraction peaks of the emulsion gels become sharper and more intense. This suggests that the addition of corn oil can partially enhance the crystallinity of the gels. This may be due to the lubrication effect intrinsic to plant oils, which reduces inter-material friction, allowing molecules to move and rearrange more easily. The infiltration of oil promotes more molecular interactions (hydrogen bonding) between SPI, KGM, and O-ChNWs in the gel matrix, thereby increasing the crystallinity of the gel. The increase in crystallinity could lead to the enhanced strength and stability of the emulsion gels.

### 3.4. The Microstructure of Emulsion Gels

**Figure 4:**
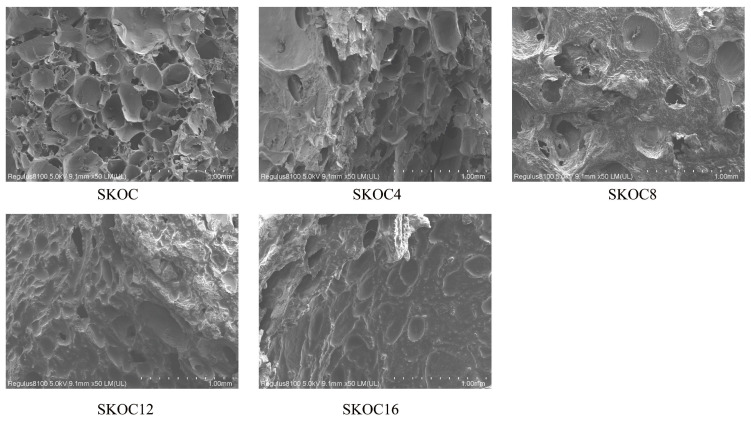
The SEM images of emulsion gels.

SEM is used to study the network structure of gels. From Figure 4, it is observed that the emulsion gels with heat-induced denatured SPI exhibit a certain mesh structure. Additionally, under alkaline conditions and heating, the random coils of KGM aggregate to form junction zones, creating a three-dimensional network. O-ChNWs act as nanofillers anchored on the SK matrix [34]. The interaction between SPI and KGM molecules enhances the structural integrity of the hydrogel. SKOC displays a regular honeycomb-like structure. The addition of 4% corn oil does not significantly change the network structure of the emulsion gel. However, with further increases in the amount of corn oil, the emulsion gel no longer exhibits a simple porous structure, as the gaps between gels are filled. Protein films are present on the surface of the fat globules, which are combined with protein filaments and the protein matrix. This indicates that the oil particles are not merely filling the three-dimensional protein network structures; instead, there are significant interactions between them. Reference. [35] proposed two main types of forces: (1) Interactions between proteins that orient them parallel along the surface of the oil, bonded by strong bonds such as disulfide bonds; (2) The interaction of the oil with oriented proteins, which involves relocating the hydrophobic groups in the protein backbone to the droplet surface, thereby achieving the adsorption of proteins at the oil–water interface. From this, it is inferred that the effects produced by oils in the gel are constructed through the synergistic actions among the oil, interfacial protein films, and proteins. In the SKOC16 gel, apparent oil droplets are exposed outside, which is due to the limited encapsulating capacity of the gel matrix, unable to accommodate an excess of corn oil.

**Figure 5:**
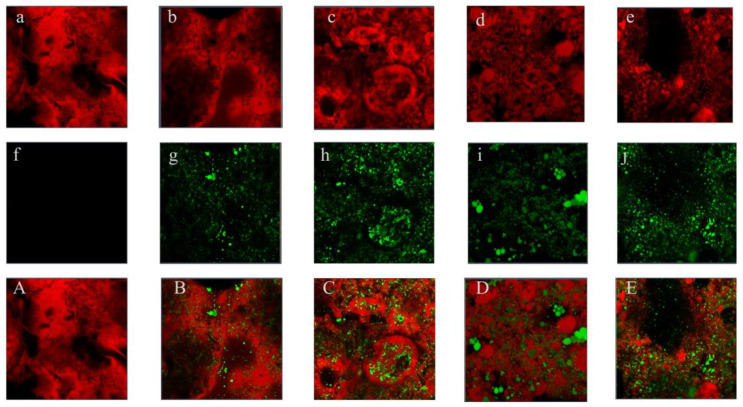
CLSM of emulsion gels. (**a**–**e** sequential fluorescence images (red) of the gel complex portion of SKOC, SKOC4-16; **f**–**j** sequential fluorescence images (green) of the oil phase portion of SKOC, SKOC4-16; **A**–**E** sequential SKOC, SKOC4-16 mixtures;).

CLSM can provide information on the distribution of oil droplets and protein components in gels [36]. The analyses depicted in Figure 5, where red and green fluorescence fields represent the SKOC and oil phase, respectively, reveal structural changes across different gel compositions. Initially, SKOC without added oil presents a discontinuous network with large pores, indicating a relatively loose structure. As the oil content increases from 4% to 12%, oil droplets evenly distribute throughout the gel, filling voids and enhancing the continuity of the network. Particularly for SKOC12, the oil droplets within the gel are small and have a more regular shape, allowing them to act as active particles that fill the gel matrix, thereby reducing the porosity of the protein network structure, promoting a tighter protein network structure, which further affects the gel’s textural properties and water-holding capacity [7,37]. However, increasing the oil content to 16% in SKOC16 introduces structural disruption. The network begins to display large voids, and oil droplets, now unevenly distributed, fail to integrate effectively into the protein network, leading to an overload phenomenon. This excess oil tends to increase the distances among network components, resulting in diminished interactions and a weakened structure.

### 3.5. WHC and OHC of Gels

**Figure 6:**
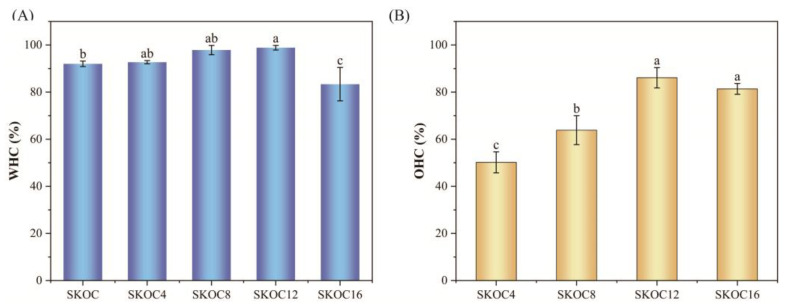
WHC (**A**) and OHC (**B**) for emulsion gels. The data are expressed as the mean ± SD (n = 6). Mean values with different letters were considered significantly different at *p* < 0.05.

WHC quantifies a gel’s ability to retain water, primarily influenced by protein–water interactions, the gel’s structural integrity, and the distribution of water throughout the gel structure [15]. WHC is a key factor affecting the quality of soy products and is closely linked to their textural characteristics. Products with a good water-holding capacity retain moisture internally, effectively reducing water loss and ensuring the overall quality of the product. The impact of oil addition on the water-holding capacity of SKOC gels is shown in Figure 6A. From Figure 6A, the SKOC emulsion gels already possess good water retention abilities, with a water-holding capacity of over 90%. As the corn oil content increases from 4% to 12%, the water-holding capacity of the gel improves. This is due to the addition of corn oil, which fills the voids in the gel matrix, making the gel network more continuous and stable. A stable three-dimensional network can enhance the WHC [38,39]. Studies have shown that the addition of a particular oil phase can improve the density of gel structures by capturing water within their pores through capillary action. Cui et al., [40] found that emulsion gels have higher WHC compared to oil-free gels. This suggests that the presence of oil droplets leads to a stronger capillary force within the gel network to retain water. However, when the oil content reaches 16%, the water-holding capacity decreases. This is due to the excessive presence of oil droplets, which increase pore sizes and reduce capillary forces, thereby disrupting the gel network’s structure and making it effectively retain water.

The OHC significantly influences the sensory properties, including taste and flavor, of food products. Figure 6B illustrates that as the corn oil content increases, the OHC of the emulsion gels initially rises and then declines. Specifically, the OHC of SKOC12 peaks at 86.10 ± 4.30%, markedly surpassing that of SKOC4 and SKOC8. This enhancement is likely attributed to the three-dimensional crosslinked network formed by SPI, KGM, and O-ChNWs, which efficiently traps liquid oil. Meanwhile, in the SPI-based gel network, corn oil acts as a filler, compacting the gel structure significantly. This compactness mirrors the effects observed when adequate moisture promotes a rigid, three-dimensional network of particles, effectively minimizing oil loss. In contrast, insufficient water fails to create capillary bridges among starch particles, leading to a weaker network and increased oil loss [41]. This deficiency likely contributes to the reduced oil-holding capacity observed in SKOC16. Moreover, excessive oil droplets may expand the distances among material components, disrupt molecular interactions, and diminish the density and uniformity of the gel. This disruption results in significant oil leakage [42].

### 3.6. Appearance and Color Analysis of Emulsion Gels

**Figure 7:**
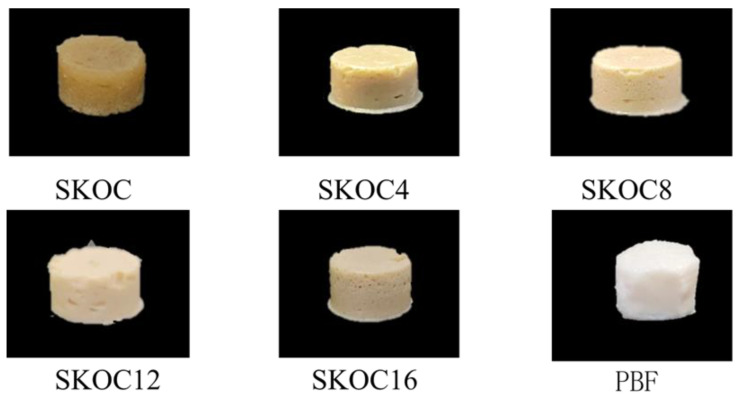
Macromorphology of emulsion gels and pork back fat (PBF).

The appearance and color measurements of gels with varying amounts of corn oil are detailed in Figure 7 and Table 1. Visually, the addition of corn oil notably influences the appearance of the gel. Specifically, SKOC gels exhibit a deeper yellow–brown color, which significantly diverges from the appearance of commercial pork back fat, resulting in less appealing sensory properties. However, the incorporation of corn oil does enhance the gel’s coloration. Using a colorimeter for more precise analysis, it was observed that as the corn oil content increased, both the L* values and whiteness of the gels also increased. This is likely due to the increased presence of oil phase and emulsion droplets on the gel’s surface, which enhance light scattering and thus elevate L* values [43]. Conversely, the b* value of the gel remains significantly higher than that of pork back fat, primarily due to the high concentration of yellow-colored SPI inherent in the gel. Similar discrepancies in b* values between experimental gels and pork back fat have been noted in other studies [40]. Interestingly, the L*, a*, and whiteness values of SKOC12 closely approximate those of commercial pork back fat. Given that color significantly impacts consumer food choices, particularly in meat products, SKOC12 holds considerable potential as a substitute for solid fats.

### 3.7. TPA of Emulsion Gels

Hardness, elasticity, cohesiveness, and chewiness are crucial in determining the texture of food, which significantly affects its palatability and acceptance. This article analyzes the textural performance of various gels compared to pork back fat, with hardness often serving as a preliminary indicator for selecting fat substitutes. According to Table 2, there is a significant difference between the gels and pork back fat in terms of hardness and chewiness. Without the addition of oil, the texture of the gels differs greatly from that of pork back fat; however, the addition of oil significantly enhances both the hardness and chewiness of the gels. Elasticity describes the ability of the gel to recover and maintain its shape after structural deformation. The study shows that the elasticity index of all gels is above 0.95, effectively mimicking that of pork back fat [28]. Cohesiveness indicates the ability of gel samples to recover after the initial compression [44]. The gels and pork back fat exhibit similar cohesiveness, both showing a high recovery rate.

**Figure 8:**
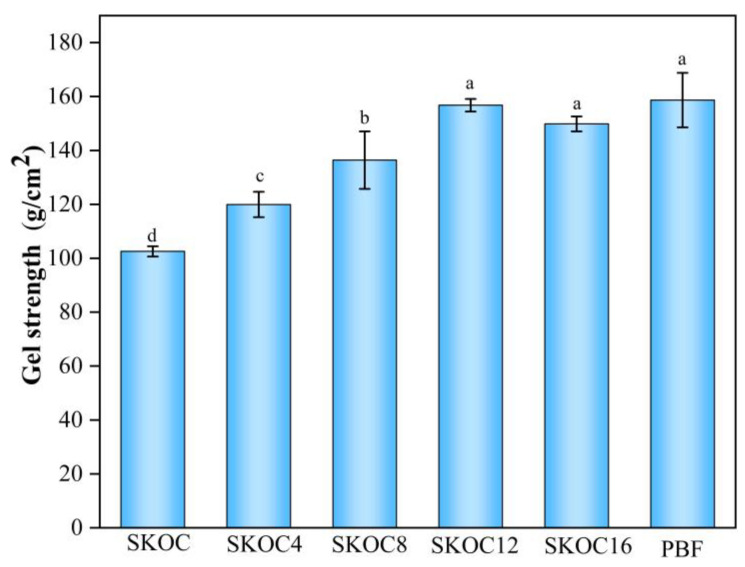
Gel strength of emulsion gels and pork back fat gels. Mean values with different letters were considered significantly different at *p* < 0.05.

Gel strength is defined as the initial force required to disrupt a gel, which reflects the integrity of the gel’s structure [45]. As shown in Figure 8, the initial gel strength increases with the addition of oil to the emulsion gel. This is because the incorporation of oil helps establish a more compact network, possibly hardening the gel structure, thus enhancing the gel strength [46]. However, the further addition of oil causes a decline in gel strength for SKOC16. This reduction results from excessive oil droplets expanding the distance between molecular substances, disrupting their interactions, and subsequently weakening the gel. Notably, the texture and gel strength of SKOC12 closely resemble those of pork back fat, underscoring its significant potential as a fat substitute.

### 3.8. Nutritional Content and Energy Value of Emulsion Gels

After comparing the physical and structural properties of gels and pork back fat, SKOC12 has shown high potential as a fat substitute. Further tests compared the nutritional content and caloric values of SKOC12 with commercially available pork back fat. As shown in Table 3, pork back fat contains 88.33 ± 0.08% fat, 7.50 ± 0.01% water, and 3.99 ± 0.05% protein. In contrast, SKOC12 has significantly different compositions, containing only 12.43 ± 0.09% fat, 69.67 ± 0.21% water, and 10.94 ± 0.06% protein. This fat substitute has a higher water content and remarkably lower fat content. Moreover, the fat content in pork back fat is 7.11 times higher than that in SKOC12 and is predominantly composed of saturated fatty acids. These fatty acids are known to elevate the risk of chronic diseases, obesity, and heart disease [47]. Conversely, using vegetable oils, which contain unsaturated fatty acids, as substitutes can reduce these health risks [48]. Moreover, SKOC12 exhibits a substantially higher protein content and drastically reduced caloric value of only 181.41 ± 0.32 kcal/100 g compared to 820.47 ± 0.46 kcal/100 g in pork back fat, making it an attractive option for populations seeking lower-calorie diets. Overall, SKOC12 not only aligns well as a textural substitute for pork back fat but brings significant nutritional enhancements, positioning it as an excellent fat substitute in dietary applications.

## 4. Conclusions

In this experiment, an emulsion gel simulating plant-based fat was prepared using corn oil, SPI, and KGM. The rheological results showed that SKOC12 exhibited the highest apparent viscosity and demonstrated frequency-dependent behavior. The water retention and oil-holding capacity results indicated that SKOC12 could effectively lock in a significant amount of moisture and stabilize oil. FT-IR and XRD analyses revealed that the addition of corn oil influenced the interactions between molecules in the gel, thereby enhancing the system’s crystallinity. The SEM and CLSM results showed that after the addition of corn oil, the gel network structure changed, with oil droplets evenly distributed within the matrix. Comparisons with the texture, gel strength, and color of pork back fat showed that SKOC12’s gel strength, hardness, chewiness, brightness, red value, and whiteness were closest to that of pork back fat. Furthermore, the basic chemical composition of SKOC12, compared to pork back fat, had a lower fat content and higher protein content, and provided less energy, making it a suitable substitute for some of the pork back fat in low-fat diets. Therefore, this preliminary study suggests that such emulsion gels have the potential to replace traditional pork back fat in low-fat meat products. However, further investigation is needed to (1) validate its safety through animal models, (2) conduct a cost–benefit analysis between plant-based and pork fat components, and (3) optimize formulations for industrial scalability.

## Figures and Tables

**Table 1:** Color of various gels and pork back fat.

	L*	A*	B*	Whiteness
SKOC	26.99 ± 0.18 ^e^	−1.19 ± 0.06 ^d^	8.27 ± 0.27 ^d^	26.48 ± 0.15 ^c^
SKOC4	29.95 ± 2.23 ^d^	−1.21 ± 0.16 ^d^	12.17 ± 0.35 ^c^	28.88 ± 2.14 ^c^
SKOC8	35.71 ± 0.43 ^c^	0.61 ± 0.07 ^c^	12.55 ± 0.51 ^c^	34.50 ± 0.41 ^b^
SKOC12	46.74 ± 1.74 ^ab^	1.75 ± 0.19 ^b^	14.62 ± 0.52 ^b^	44.74 ± 1.66 ^a^
SKOC16	48.57 ± 1.68 ^a^	1.88 ± 0.12 ^ab^	17.39 ± 0.56 ^a^	45.67 ± 1.41 ^a^
PBF	45.34 ± 2.24 ^b^	2.01 ± 0.16 ^a^	4.58 ± 0.54 ^e^	45.13 ± 2.19 ^a^

Note: Mean values with different letters were considered significantly different at *p* < 0.05.

**Table 2:** TPA parameters of various gels and pork back fat.

Scheme.	Hardness (N)	Springiness (mm)	Cohesiveness (−)	Chewiness (mJ)
SKOC	545.29 ± 6.84 ^d^	0.96 ± 0.01 ^a^	0.86 ± 0.03 ^a^	460.27 ± 5.22 ^e^
SKOC4	626.53 ± 39.19 ^c^	0.96 ± 0.02 ^a^	0.85 ± 0.03 ^a^	504.23 ± 17.47 ^d^
SKOC8	676.83 ± 29.09 ^b^	0.96 ± 0.02 ^a^	0.85 ± 0.04 ^a^	549.27 ± 3.84 ^c^
SKOC12	768.07 ± 19.65 ^a^	0.98 ± 0.01 ^a^	0.86 ± 0.04 ^a^	684.38 ± 42.47 ^a^
SKOC16	746.53 ± 17.24 ^a^	0.98 ± 0.02 ^a^	0.87 ± 0.06 ^a^	613.12 ± 8.15 ^b^
PBF	761.13 ± 19.67 ^a^	0.98 ± 0.01 ^a^	0.84 ± 0.04 ^a^	686.16 ± 23.37 ^a^

Note: Mean values with different letters were considered significantly different at *p* < 0.05.

**Table 3:** Nutrient content and energy content of SKOC12 and pork back fat.

	PBF	SKOC12
Fat (%)	88.33 ± 0.08	12.43 ± 0.09
Moisture (%)	7.50 ± 0.01	69.67 ± 0.21
Protein (%)	3.99 ± 0.05	10.94 ± 0.06
Ash (%)	0.11 ± 0.00	1.24 ± 0.01
Energy (kcal/100 g)	820.47 ± 0.46	181.41 ± 0.32

## Data Availability

The original contributions presented in this study are included in the article. Further inquiries can be directed to the corresponding author.
